# Understanding correlates of child stunting in Ethiopia using generalized linear mixed models

**DOI:** 10.1186/s12889-019-6984-x

**Published:** 2019-05-22

**Authors:** Kasahun Takele, Temesgen Zewotir, Denis Ndanguza

**Affiliations:** 1 African Center of Excellence in Data Science, University of Rwanda, Kigali, Rwanda; 2 School of Mathematics, Statistics and Computer Sciences, University of KwaZulu-Natal, Durban, South Africa; 3 College of Science and Technology, University of Rwanda, Kigali, Rwanda

**Keywords:** Random effect, Cluster effect, GLMM, Under five, AIC

## Abstract

**Background:**

Stunting is an indicator of the devastating result of malnutrition in early childhood. The effects of childhood stunting are irreparable physical and cognitive harm. It is an issue of the great public health importance throughout Sub-Saharan African countries including Ethiopia. Therefore, identification of the risk factors of child stunting from recent data is very important for timely intervention.

**Methods:**

The 2016 Ethiopian Demographic and Health Survey data were used for this study. A generalized linear mixed model which is an extension of the general linear model was employed to identify socioeconomic, demographic, environmental and health related risk factors for stunted under-five children.

**Results:**

The result shows that the age and sex of the child, preceding birth interval, mother’s body mass index, household wealth index, mother’s education level, breastfeeding period, type of toilet facility, use of internet and source of drinking water were the major determinants of stunting of under-five children in Ethiopia.

**Conclusion:**

The study indicated that children from undernourished mothers, who are not breastfeeding, from poor households, households that have no toilet facilities, who are male, older age (between 12 to 59 months), who have illiterate mother and short birth spacing were associated with stunting problems. Therefore, family planning education and policy is required for the country to improve on under-five age stunting problems.

## Background

Stunting is an indicator of the devastating result of malnutrition in early childhood and is strongly associated with numerous short term and long-term conditions. These conditions include increased morbidity and mortality, delayed growth, poor children’s wellbeing and social inequalities and long-term educational and economic consequences [1–4]. The effects of childhood stunting also include irreparable physical and cognitive damage. It is a scourge of early beginnings and has far-reaching consequences. According to a Unicef report, in 2017, among the indicators of childhood malnutrition, stunting was found to be the most widespread affecting an estimated 151 million children worldwide [5]. Furthermore, the report reveals that there is insufficient progress to reach the World Health Assembly target set for 2025 to attain a 40% reduction of stunting. Unexpectedly, the number of stunted children has risen from 50.6 million to 58.7 between 2000 and 2017 in Africa [5]. Due to this rise in the past years, stunting is considered an issue of great public health importance throughout developing countries particularly in Sub-Saharan African countries. As a result, in its national nutrition strategy and plan, Ethiopia has given a priority to reduce childhood stunting particularly for children under two years. This can be done by targeting pregnant and lactating mothers [6]. Despite concrete progress made against childhood malnutrition in the past decade, Ethiopia remains a tremendously malnourished country. However, the trend shows that the prevalence of stunting has decreased from 58% in 2000 to 38% in 2016, which is an average decline of more than 1% annually, but the problem is still high. This improvement has also been slightly uneven across the country. According to the 2016 Ethiopian Demographic and Health Survey report, variation in rates of childhood stunting range from low in Addis Ababa (15%) to very high in regions of Amhara, Benishangul, Afar, and Dire Dawa (41–46%). This shows that up to the present time, the country has fallen behind expectations with regards to addressing stunting prevalence.

As a result, there is a need to identify factors, in a local context, to allow a successful formulation of a national malnutrition intervention policy. Furthermore, some of previous studies have indicated that socioeconomic, demographic and environmental and health related factors could cause childhood stunting worldwide [3, 7, 8, 9, 10, 11]. For instance, [8, 9] child age and sex as non-modifiable determinants and household wealth, maternal education and family size were identified as crucial protective determinants of stunting. Similarly, another study reported that household and family factors, inadequate complementary feeding, inadequate breastfeeding practices, and infections are the most important contributing factors for stunted growth and development [10]. Furthermore, some of previous studies in Ethiopia show a wide variety of risk factors that include socioeconomic, environmental, demographic and other factors associated with stunting [7, 12], but most of these studies fail to account for possible variability between the primary sampling units.

To accommodate this source of variability, we use generalized linear mixed model that includes possible random effects. In addition, there is still a great need to identify important factors from recent data for a successful formulation of a national malnutrition intervention strategy. Therefore, the aim of this study is to investigate the relationship between the stunting status of under-five children and selected socio-economic, demographic and environmental factors.

## Methods

### Data source

This study uses the 2016 Ethiopian Demographic Health Survey (EDHS) data. One of the central objectives of the DHS is to provide an up-to-date information of childhood malnutrition. The survey drew a representative sample of women of reproductive age using a two-stage cluster design and the enumeration areas were the sampling units for the first stage. The sample included 645 enumeration areas among 202 in urban areas and 443 in rural areas. Of the total 15,683 women, 5348 from urban and 10,335 from rural households successfully completed the interview. Among the sampled households, data for 8743 children under the age of five were used. The analysis presented in this study is based on under-five age children with growth retardation or stunting as the response variable. The independent covariates considered are socioeconomic, demographic, household, child and spatial characteristics. Sex of child, age of child, preceding birth interval, mother’s body mass index, household wealth index, source of drinking water, type of toilet facility, breastfeeding, mother’s education level and region were included as determinants.

### Generalized linear mixed model (GLMM)

Generalized linear mixed models are a powerful class of statistical models that combine the characteristics of generalized linear models and mixed models (include both fixed and random predictor variables). Generalized linear mixed models are an extension of the class of generalized linear models in which random effects are included to the linear predictors. This allows the modeling of correlated, possibly no-normally distributed data with flexible accommodation of covariates. This can overcome the modeling problem of over-dispersion in the data and at the same time, accommodate the population heterogeneity. Recently, GLMMs have become important tools for clustered data, particularly in public health.

#### Specification of a GLMM

Let *Y*_*ij*_ be the *j*th binary response measured for cluster i, i = 1, 2, … , N, j = 1, 2, … , n_i_ and Y_i_ is the n_i_-dimensional vector of all measurements available for cluster *i*, conditionally on *q*-dimensional random effects ***b***_***i***_ and assumed to be drawn independently from the *N*(**0**, ***D***). Then, the responses *Y*_*ij*_ are independent with densities of the form [13].

1 $$ {\boldsymbol{f}}_{\boldsymbol{i}}\left({y}_{ij}/{\boldsymbol{b}}_{\boldsymbol{i}},\boldsymbol{\beta}, \phi \right)=\mathit{\exp}\left\{{\phi}^{-1}\left({y}_{ij}\left({\theta}_{ij}\right)-\psi \left({\theta}_{ij}\right)\right)+c\left[{y}_{ij},\phi \right]\right\} $$

With $g\left({\mu}_{ij}\right)=g\left[E\left({y}_{ij}/{\boldsymbol{b}}_{\boldsymbol{i}}\right)\right]={x}_{ij}^{\prime}\beta +{z}_{ij}^{\prime }{\boldsymbol{b}}_{\boldsymbol{i}}$**,** for a known link function *g*(.), ***x***_***ij***_ is the *i*^*th*^ row of the matrix for the fixed effects, ***z***_***ij***_ is the i^th^ of the row matrix for the random effects, **β** is the parameter vector of unknown fixed effect, ***b*** is a parameter vector of random effect and ***b*** ∼ **N**(**0**, **D**)***,*** **ψ** is a scale parameter and **θ** is the natural parameter. Under this GLMMs setting, the logit link function is defined as

2 $$ g\left({\mu}_{ijk}\right)= logit\left({\mu}_{ijk}\right)=\mathit{\log}\left[\frac{\mu_{ijk}}{1-{\mu}_{ijk}}\right]={\eta}_{ijk} $$

Where the conditional expectation of *μ*_*ij*_ = *E*(*y*_*ijk*_/*b*_*i*_, *x*_*i*_) = *p*(*y*_*ijk*_/*b*_*i*_, *x*_*ijk*_). This model can be expressed as

3 $$ p\left({\mathrm{y}}_{ijk}/{b}_i,{x}_{ijk},{z}_{ijk}\right)={g}^{-1}\left({\eta}_{ijk}\right) $$

Where *g*^−1^(*η*_*ijk*_), is a logistic cumulative distribution function and defined as *g*^−1^(*η*_*ijk*_) = [1 + exp(−*η*_*ijk*_)]^−1^. The logistic distribution simplifies parameter estimation due to fact of probability density function is related to the cumulative density function [14].

Furthermore, as introduced in [13], fitting of GLMM is mainly based on maximum likelihood principles and as a result, inferences for the parameters are obtained from classical maximum likelihood theory. Indeed, assuming that the fitted model is appropriate, the obtained estimators are asymptotically normally distributed with the correct values as means with the inverse Fisher information matrix as the covariance matrix. Then, the Wald-type test for comparing standardized estimates to the standard normal distribution can be used.

## Results

### Exploratory data analysis

The result of cross-tabulation analysis for under-five stunted children is summarized in Table 1. *P*-value indicated in Table 1 is obtained from chi-square test. Cross-tabulation analysis reveals that the current age of child, sex of child, preceding birth interval, mother’s body mass index, household wealth index, mother’s educational level, breastfeeding, type of toilet facility, source of drinking water and use of the internet are significantly associated with childhood stunting at the 5% level of significance. Table 1 indicates that there is high prevalence of stunting among children of the age group between 24 to 59 months and 12 to 23 months, which are 44.1 and 38.5% respectively (*p* − *value <* 0*.*000). Moreover, from Table 1, male children are more likely to be stunted when compared to their female counterparts (38%, *p* − *value <* 0*.*000). Children from poor households are more exposed to the stunting problem (41.6%). Furthermore, breastfeeding is associated with stunting (*p* − *value <* 0*.*001). The prevalence of stunting is higher among non-breastfeeding children than breastfeeding children (38.2%). The literacy of the mother is highly associated with childhood stunting (*p* − *value <* 0*.*000). Children with illiterate mothers are more vulnerable to stunting (40.3%) than those with literate mothers. In addition, children born with short birth spacing are more exposed to stunting than those with long birth spacing in Ethiopia (37.7%, p − *value <* 0*.*000). Similarly, use of the internet is highly associated with childhood stunting (*p* − *value <* 0*.*000).

**Table 1:** Distribution of childhood stunting and its associated selected factors

Covariates	Level	Stunting N (%)		*P*-value
		Not stunted	Stunted	
Sex of child	Male	2760(62)	1695 (38)	0.001
	Female	2803 (65.4)	1485 (34.6)	
Current age of child in months	0–11 months	1620 (86.9)	244 (13.1)	0.000
	12–23 months	1086 (61.5)	679 (38.5)	
	24–59 months	2857 (55.9)	2257 (44.1)	
Preceding birth interval (months)	less than 24 months	2242 (62.8)	1329 (37.2)	0.000
	24–47 months	2156 (62.3)	1307 (37.7)	
	48 and above months	1165 (68.2)	544 (31.8)	
Mother’s BMI	greater than or equal to18.5	1266 (59.0)	878 (41.0)	0.000
	less than 18.5	4297 (65.1)	2302 (34.9)	
Wealth index combined	Poor	2726 (58.4)	1939 (41.6)	0.000
	Middle	789 (62.5)	473 (37.5)	
	Rich	2048 (72.7)	768 (27.3)	
Source of drinking water	piped	939 (77.6)	271 (22.4)	0.000
	public tap	2439 (61.3)	1543 (38.7)	
	protected spring	421 (60.8)	271 (39.2)	
	other	1764 (61.7)	1095 (38.3)	
Currently breastfeeding	No	1784 (61.8)	1104 (38.2)	0.011
	Yes	3779 (64.5)	2076 (35.5)	
Type of toilet facility	flush toilet	286 (81.7)	64 (18.3)	0.000
	latrine	2917 (65.8)	1515 (34.2)	
	no facility	2360 (59.6)	1601 (40.4)	
Highest educational level	No education	3325 (59.7)	2241 (40.3)	0.000
	Primary	1499 (66.2)	766 (33.8)	
	Secondary and higher	739 (81.0)	173 (19.0)	
	No	5346 (62.9)	3156 (37.1)	
Use of internet	Yes	217 (90.0)	24 (10.0)	0.000

### Results from generalized linear mixed model (GLMM)

Model selection was attained by first including all covariates into the model and then evaluating whether or not interaction terms needed to be incorporated. All covariates and their interactions were fitted into the model at the same time and only those that were statistically significant were retained in the final model. The final selected model for child growth retardation contained eight main effects and two two-way interaction effects as shown in eq. (4).

4 $$ logit\left({\mu}_{ij}\right)={\beta}_0+{\beta}_1 child\ {age}_{ij}+{\beta}_2 child\ {sex}_{ij}+{\beta}_3{birth\ interval}_{ij}+{\beta}_4{mother\ education}_{ij}+{\beta}_5 mother\ {BMI}_{ij}+{\beta}_6{wealth\ index}_{ij}+{\beta}_7{breastfeeding}_{ij}+{\beta}_8{toilet\ facility}_{ij}+{\beta}_9 child\  age\ast birth\ {interval}_{ij}+{\beta}_{10} mother\  BMI\ast source\ {of\ water}_{ij}+{b}_i+{b}_{ij} $$

where, *β*_1_, *β*_2_, … , *β*_10_ are the unknown parameter coefficients of fixed effects, *b*_*i*_ *and b*_*ij*_ are regional and cluster level random intercepts respectively.

### Results and interpretations

The ratio of generalized chi-square statistic is 0.98 which is close to one (Table 2). This is the measure of the residual variability in the marginal distribution of the data. Since the value is close to one, the variability in the data has been properly modelled and there is no residual over-dispersion. As shown in Table 2, the AIC of the two random intercept model is 10,337.5. This is a reduction from 10,419.0 which is the AIC for the one random intercept model. The small *p*-value of the log likelihood ratio test (*P <* 0*.*001) indicates that the two random intercept model is the parsimonious one. Similarly, from the same table, the ratio of the generalized chi-square statistic is 0.98.

**Table 2:** Information criteria for the comparison of two random intercept models

Model	AIC	BIC	LogLink	Deviance	*P*-value
One RIM	10,419.0	10,560.5	− 5189.5	10,379.0	
Two RIM	10,337.5	10,486.1	− 5147.8	10,295.5	0.000

Generalized chi square/DF = 0.98

Table 3 indicates that the child age, sex of child, preceding birth interval, mother’s body mass index, wealth index, mother educational level, breastfeeding, type of toilet facility, use of internet, interaction effect of child age and preceding birth interval and mother’s body mass index and source of drinking water were found to significantly affect child stunting.

**Table 3:** Type III analysis of effects for the Generalized Linear Mixed Model

Effect	DF	F-value	*P*-value
Child age	2	209.83	< 0.001
Sex of child	1	11.66	< 0.001
Preceding birth interval	2	0.74	0.479
Mother’s body mass index(BMI)	1	11.87	< 0.001
Wealth index	2	16.53	< 0.001
Mother education level	2	9.86	< 0.001
Breast feeding	1	13.46	< 0.001
Types of Toilet facility	2	3.26	0.038
Use of Internet	1	12.90	< 0.001
Source of drinking water	3	1.46	0.480
Child age: Preceding birth interval	4	2.79	0.025
BMI: Source of drinking water	3	3.58	0.035

Table 4, presents odds ratio estimates associated with age of child, sex of child, preceding birth interval, mother’s body mass index, household’s wealth index, mother’s educational level, breastfeeding, type of toilet facility, use of internet and source of drinking water. The results indicate that stunting is higher for children aged between 24 and 59 months (OR = 7.479, *P* <  0.001; 95% CI: 1.746, 2.228) and 12–24 months (OR = 5.556, P <  0.001; 95% CI: 1.413, 2.001) when compared to the younger children (0–11 months). Based on the results, female children are 16% less likely to be stunted than male children. With reference to households with flush toilet facilities, stunting is high for children from households with no such toilet facilities (OR = 1.523, *P* = 0.012; 95% CI: 0.064, 0.726) followed by those with latrines (OR = 1.405, *P* = 0.038; 95% CI: 0.006, 0.638). From Table 4, it is indicated that mother’s educational level is significantly associated with the growth of children under five. Furthermore, it revealed that children from a mothers with primary education, and those with secondary or higher educational levels are 13.1 and 36.2%, respectively, less likely to be stunted than children from mothers with no formal education. As it can be seen from Table 4, household’s economic status significantly affects the growth of a child. In addition, it is observed that children from middle-income households (OR = 0.791, *P* = 0.0034; 95% CI: − 0.399, − 0.084) and rich households (OR = 0.648, *P* <  0.000; 95% CI: − 0.571, − 0.256) are less likely to be stunted than children from poor households. From Table 4, it is observed that children who were not breast-fed are 1.225 times more likely to get affected by stunting than children who were breastfeed. Children from the households use internet are 56.7% less likely to get affected by stunting.

**Table 4:** Parameter estimates of main effects for stunting of children using GLMM

Covariates	Estimates	S.E	95% CI	OR	P-value
Intercept	−2.16258	0.24340	(−3.380, −2.002)	0.115	< 0.001 ***
Child age (ref = 0–11 months)					
12–23 months	1.71504	0.15198	(1.413, 2.001)	5.556	< 0.001 ***
24–59 months	2.01208	0.13679	(1.746, 2.228)	7.479	< 0.001 ***
Preceding birth interval(ref = < 24)					
24–47 months	0.02248	0.16779	(−0.307, 0.351)	1.023	0.893
48 and above months	0.29244	0.18766	(− 0.055, 0.680)	1.34	0.119
Sex of child (Female)	− 0.17457	0.04912	(−0.271, − 0.079)	0.84	< 0.001 ***
BMI (less than 18.5)	− 0.20876	0.05841	(−1.064, − 0.200)	0.812	< 0.001 ***
Wealth index (ref = poor)					
Middle	−0.23438	0.08017	(−0.399, − 0.084)	0.791	< 0.001 ***
Rich	− 0.43440	0.07681	(−0.571, − 0.256)	0.648	< 0.001 ***
Mother education (ref = no education)					
Primary	−0.14040	0.06230	(−0.251, − 0.006)	0.869	0.024 *
Secondary and higher	−0.44999	0.11318	(− 0.636, − 0.186)	0.638	< 0.001 ***
Breast (No)	0.20309	0.05585	(0.093, 0.312)	1.225	< 0.001 ***
Types of toilet facility (ref = flush)					
Latrine	0.33974	0.16403	(0.006, 0.638)	1.405	0.038 *
No facility	0.42179	0.16824	(0.064, 0.726)	1.523	0.012*
Use of internet (Yes)	−0.83650	0.24302	(−1.289, − 0.332)	0.433	< 0.001 ***
Source of drinking water (ref = piped)					
Public tap	00.17173	0.17386	(−0.668, 0.219)	1.187	0.323
Protected spring	0.05793	0.24192	(−0.903, 0.165)	1.059	0.810
Other	0.29269	0.17915	(−0.608, 0.311)	1.340	0.102

Significance codes: 0 ‘***’ 0.001 ‘**’ 0.01 ‘*’ 0.05

### Interaction effects

**Fig. 1:**
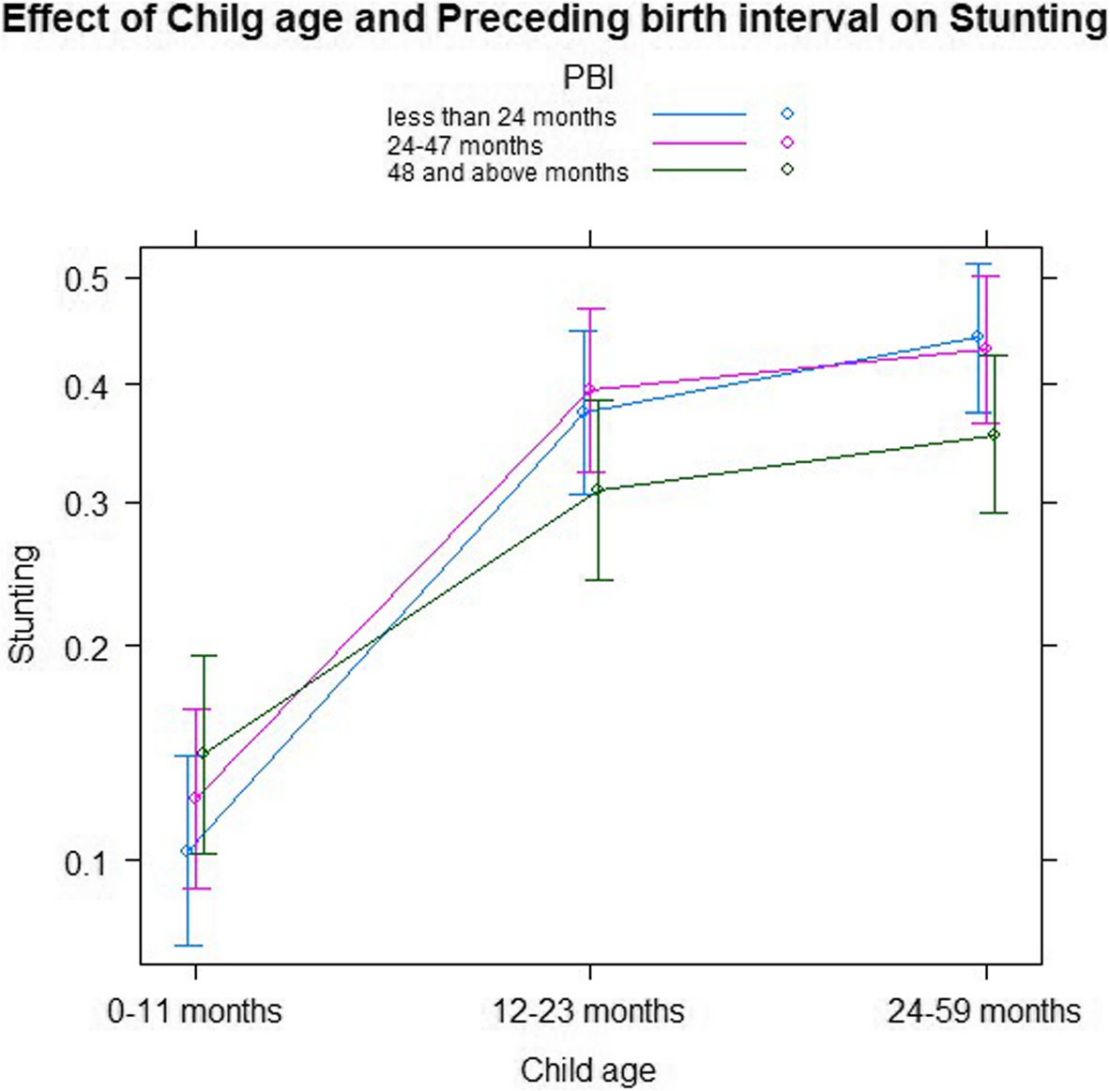
Log odds associated with stunting of children by preceding birth interval and children age

**Fig. 2:**
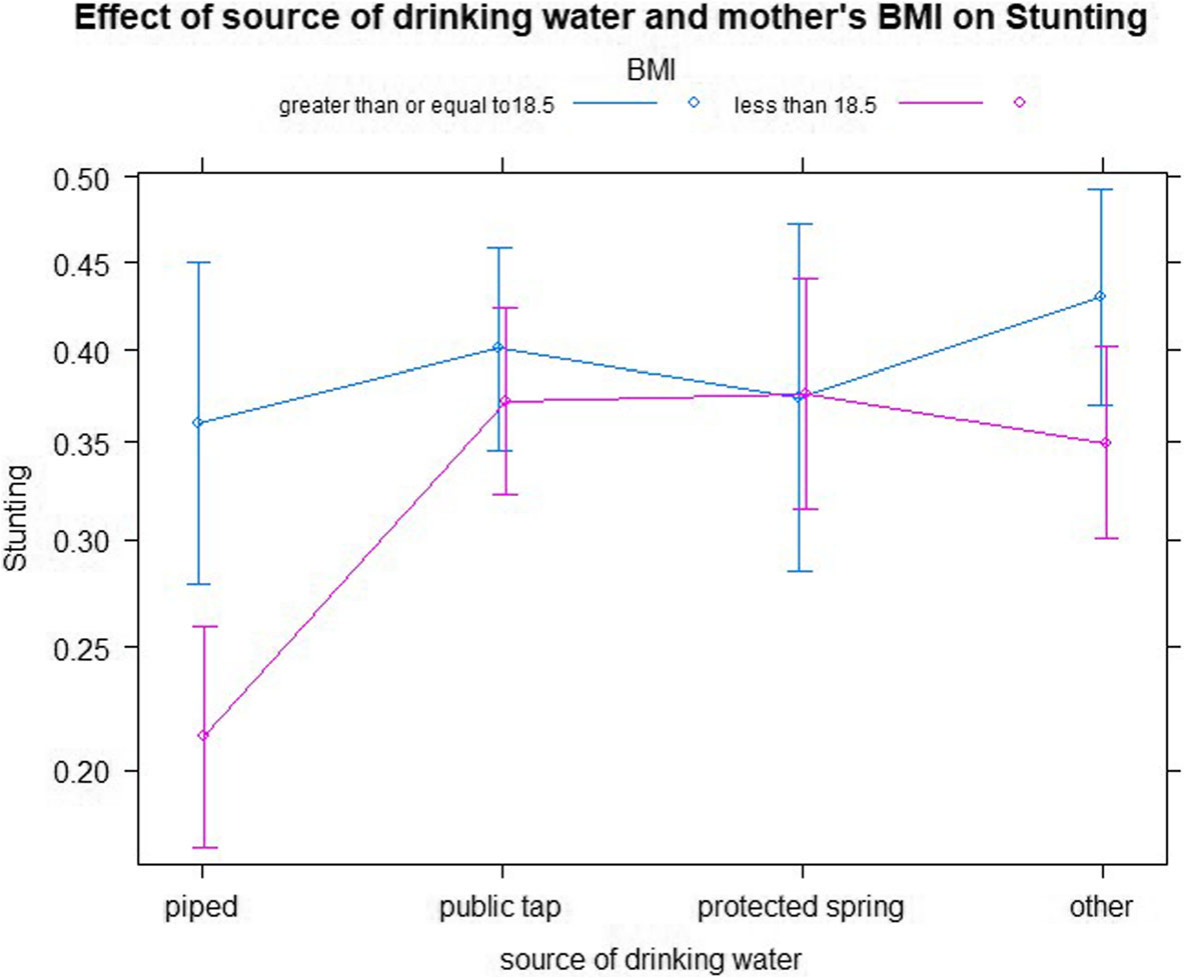
Log odds associated with stunting of children by source of drinking water and mother’s body mass index

The relationship between preceding birth interval (less than 24 months, 24–48 months and above 48 months) and age of child (0–11 months, 12–23 months and 24–59 months) is presented in Table 5 and Fig. 1. The figure shows all data points, with stunting plotted against child age group. As the result indicates, children with preceding birth interval of 48 months and above are less likely to be stunted when compared to children with preceding birth interval less than 24 months in all age groups (OR = 0.540, OR = 0.535, *P*-value< 0.0028, 0.0074 respectively). Furthermore, there is a relationship between sources of drinking water (tap, protected, spring and others) and mother’s body mass index (less than 18.5 and above 18.5) (Table 5). Figure 2 shows all data points with stunting plotted against source of drinking water and mothers body mass index. Children from households using public tap water, protected spring water and other sources of water are more likely to be stunted compared to households that use piped water in all mothers body mass index group. The same is portrayed in Table 5.

**Table 5:** Parameter estimates with two-way interaction effects for stunting of children using GLMM

Covariates	Estimates	S.E	95% CI	Odds ratio	P-value
Age and Preceding birth interval (Ref = 0–11 months: PBI less 24)					
12–23 months:PBI24–47 months	−0.07958	0.20433	(−0.468, 0.334)	0.923	0.696
24–59 months:PBI24–47 months	− 0.16060	0.17972	(− 0.511, 0.194)	0.852	0.371
12–23 months:PBI48 and above months	−0.62538	0.23360	(−1.086, − 0.169)	0.535	0.007 **
24–59 months:PBI48 and above months	−0.61601	0.20596	(−1.034, − 0.227)	0.540	0.002**
Source of drinking water and mother’s BMI (Ref = ≥ 18.5 BMI: Piped water)					
Less than 18.5: public tap	0.61155	0.19454	(0.171, 1.001)	1.843	0.001**
Less than 18.5: protected spring	0.74230	0.26869	(0.174, 1.321)	2.101	0.005**
Less than 18.5: other	0.39204	0.20050	(0.057, 0.799)	1.478	0.050*
Random effects	Variance	SD			
Cluster	0.2190	0.4680			
Region	0.1054	0.3247			
Residual	1.11	1.05			

Significance codes: 0 ‘***’ 0.001 ‘**’ 0.01 ‘*’ 0.05

## Discussion

In this study, the generalized linear mixed model employed to investigate all possible determinants of child stunting and to capture the interconnectedness among various risk factors within the model. The study used the 2016 EDHS and R version 3.5.2 for the analysis of the data. Observing more carefully within groups of determinants, our analysis confirms the importance of age of child and sex of child as non-modifiable risk factors and preceding birth interval, mother’s body mass index, household wealth index, mother’s education level, breastfeeding, type of toilet facility, use of internet and source of drinking water as major statistically significant risk factors. In Ethiopia, approximately 38% of children under the age of five are too short for their age.

The results shows that the age of child is positively associated with the child’s stunting. Children aged between 24 to 59 months and 12 to 23 months are at a higher risk of stunting than those whose ages are between 0 to 11 months. Similar to studies carried out [7–9], this study confirmed that older children were associated with a higher risk of stunting. Similarly, based on demographic factors associated with stunting, our analysis showed that male children are at a higher risk of stunting than their female counterparts. Furthermore, the findings indicated that children with preceding birth interval of 48 months and above are less likely to be stunted as compared to preceding birth interval less than 24 months in all age groups. The results of this study support the results from Stewart, C. P., et al. (2013) and Dewey, K. G. and R. J. Cohen (2007 [10, 15] which shows that short birth spacing increases the risk for depleted maternal reserves in subsequent pregnancies, with negative consequences for both mother and child. In addition, the study found that household wealth index is an important socioeconomic factor that affects the growth of children in Ethiopia, as it is associated with negative effects on the child stunting. Everywhere, the poor suffer poor diet, inadequate schooling, poor clothing, poor hygiene and health. This indicates that children in poor households are at high risk of stunting problems when compared to children from rich households. The findings regarding the household wealth is concur with those of [7, 16, 17]. The mother’s educational level is an important risk factor associated with stunting of child. Children from illiterate mothers are more vulnerable to the risk of stunting than children from mothers who attainted at least primary education. This result is in agreement with the previous findings [9, 10]. The attainment of higher education by mothers can improve the understanding and respond to the nutrition behavior changes of their children. Furthermore, our analysis revealed that access to improved toilet facilities are associated with the reduced risk of stunting in children. This implies that stunting is high among children from households with no toilet facilities. The type of toilet facilities used by a household is an indicator of household wealth as poor households are less likely to have sanitary toilet facilities. This finding is in line with results of Dearden, K. A., et al. (2017) and Khatab, K. (2010) [18, 19]. The study found that mother’s body mass index is (nutritional status) negatively associated with stunting of child.

## Conclusion

Based on the generalized linear mixed model, this paper identified the determinants of stunting of children in Ethiopia. The result showed that the age of child, sex of child, preceding birth interval, mother’s body mass index, household wealth index, mother’s educational level, breast-feeding, type of toilet facilities, use of internet and source of drinking water are the correlates of stunting of children in Ethiopia. Children from undernourished mothers, non-breastfed, from poor households, from households that have no toilet facilities, who are male, who are older (between 12 to 59 months) are associated with stunting problems. The results of our analysis suggested that children from uneducated mothers are at higher risk of stunting problems in Ethiopia. As a result, to reduce the risk of childhood stunting in the country, the mothers literacy program should be encouraged. Furthermore, it was found that children within less than 24 months of preceding birth interval are at high risk of stunting. Therefore, family planning education and policy is required in the country to improve on under-five age stunting problems of children.
